# Wildlife as a Potential Reservoir of Paratuberculosis: Molecular Evidence of *Mycobacterium avium* Subspecies *paratuberculosis* in a Biosphere Reserve, the Sudan

**DOI:** 10.3390/ani16203216

**Published:** 2026-10-13

**Authors:** Enass M. Abdalla, Omer M. Meina, Sanaa M. Idris, Wisal A. Elmagzoub, ElSagad Eltayeb, Julius B. Okuni, Lonzy Ojok, Mohamed E. Mukhtar, Behrouz Alizadeh Savareh, Paula E. Schweizer, Ahmed Abd El Wahed, Aisha E. Faki, Ahmed A. Gameel, Uwe Truyen, Kamal H. Eltom

**Affiliations:** 1 Institute of Animal Hygiene and Veterinary Public Health, Faculty of Veterinary Medicine, University of Leipzig, An den Tierkliniken 1, D-04103 Leipzig, Sachsen, Germany; enass.mukhtar_abdalla_mohammed@uni-leipzig.de or enassmukh17@gmail.com (E.M.A.); sanaaidris15@gmail.com (S.M.I.); wisalelmagzoub@gmail.com (W.A.E.); alizade.behruz@gmail.com (B.A.S.); p.schweizer@vetmed.uni-leipzig.de (P.E.S.); truyen@vmf.uni-leipzig.de (U.T.); 2 Department of Animal Health and Safety of Animal Products, Institute for Studies and Promotion of Animal Exports, University of Khartoum, Khartoum North 13314, Khartoum, Sudan; 3 Wildlife Research Centre, Animal Resources Research Corporation, Ministry of Animal Resources Corporation, Khartoum North 13319, Khartoum, Sudan; omar1313ifr@gmail.com (O.M.M.); aishelfaki@hotmail.com (A.E.F.); 4 Department of Pathology, Faculty of Veterinary Medicine, University of Khartoum, Khartoum North 13314, Khartoum, Sudan; aargameel@hotmail.com; 5 Department of Biology and Biotechnology, College of Applied and Industrial Sciences, University of Bahri, Khartoum North 13312, Khartoum, Sudan; 6 Ibn Sina Specialized Hospital, Faculty of Medicine, Al Neelain University, Khartoum 11121, Khartoum, Sudan; sagadgady@yahoo.com; 7 College of Veterinary Medicine, Animal Resources and Biosecurity (COVAB), Makerere University, Kampala P.O. Box 7062, Uganda; julius.okuni@mak.ac.ug (J.B.O.); lonzyo@yahoo.com (L.O.); 8 Department of Pathology, Faculty of Medicine, Gulu University, Gulu P.O. Box 166, Uganda; 9 Department of Agricultural Extension and Rural Development, Faculty of Agriculture, University of Khartoum, Khartoum North 13314, Khartoum, Sudan; elgadidwas@yahoo.com

**Keywords:** paratuberculosis, Dinder, RAA, buffalo, oribi, reedbuck, warthog, waterbuck

## Abstract

*Mycobacterium avium* subspecies *paratuberculosis* (MAP) causes paratuberculosis (PTB), a chronic wasting enteritis of domestic and wild ruminants. Although the disease and/or its aetiology have been reported in livestock in the Sudan, they have not been investigated in wild animals. This study investigated MAP DNA prevalence in the Dinder National Park (DNP) to provide baseline data on PTB in wildlife. Fresh faecal samples (*n* = 274) of wild ruminants were collected from the ground around the six major water ponds of the DNP (44–48 samples/pond). Faecal DNA was extracted using a rapid extraction protocol and tested for MAP DNA using a real-time recombinase-aided amplification (RAA) assay. The vegetation structure around the ponds was observed and described. MAP DNA detection rates around the ponds ranged between 64.4% and 22.9%, with an overall apparent prevalence of 37.9% and a sensitivity/specificity-adjusted sample positivity of 42.4%. The faecal samples belonged to Reedbuck (*Redunca redunca*), Warthog *(Phacochoerus aethiopicus*), Waterbuck (*Kobus defasa*), African Buffalo (*Syncerus caffer*), Oribi (*Ourebia ourebia*), and among which MAP DNA detection rates were 39.4% (28/71), 39.1% (18/46), 38.0% (27/71), 37.5% (12/32) and 35.2% (19/54), respectively. The vegetation pattern around the ponds showed a notable difference that was consistent with MAP DNA detection. These results are consistent with the potential role of wildlife in paratuberculosis maintenance and transmission. Future investigations, including genotyping, would identify possible cross-transmission of the pathogen at the wildlife-domestic animal interface.

## Simple Summary

Paratuberculosis is considered a neglected disease in Africa, affecting domestic and wild ruminants (e.g., Waterbuck) and occasionally non-ruminants (e.g., Warthogs). The disease has a significant impact on wildlife conservation, particularly of endangered species. In the Sudan, the disease has been reported in domestic ruminants, but not in wild animals. This study presents the first report of evidence of *Mycobacterium avium* subspecies *paratuberculosis* in wild animals and, to the best of our knowledge, one of the first surveys for the disease in wildlife in Africa. The study area is located within the Dinder National Park, one of Africa’s largest biosphere reserves, which hosts a wide diversity of wild species. It borders the Ethiopian Alitash National Park and is connected to three Sudanese states. A high disease prevalence is reported here, making it a potential area for pathogen exposure, especially during the dry season when animal movement increases. Accordingly, a rapid molecular technique was used to assess the presence of pathogen DNA in the faeces of five wild mammals. Our results indicate a potential risk of pathogen exposure through environmental contamination with pathogen-containing faeces. Given the ecological and conservation value of wildlife, further studies are needed to better understand the prevalence and distribution of the disease among other wildlife species in the park. Such investigations would provide valuable information to strengthen disease surveillance and support effective wildlife conservation programmes.

## 1. Introduction

*Mycobacterium avium* subspecies *paratuberculosis* (MAP) is an obligate intracellular bacterium that causes paratuberculosis (PTB), most commonly known as Johne’s disease (JD). PTB is an incurable chronic granulomatous enteritis of a wide range of domestic and wild ruminants, and some non-ruminant species [1]. Animals are mostly infected at an early age, but clinical signs typically take years to emerge [2]. The disease takes from 2 to 10 years to manifest [3]. It is described in four stages: silent, subclinical, clinical, and advanced clinical, with two distinct types of MAP shedding: progressive and non- progressive [4]. The infection is mainly transmitted via the oral-faecal route, through contaminated milk, pastures, soil, water and surfaces [5]. Cross-species transmission has been reported [6], and wild animals are not an exception: they may intermittently shed MAP and contribute to disease transmission to livestock [7]. In areas where wild animals interact with farmed livestock [2], several studies have reported that genetically similar MAP isolates originating from such animals are found in both farms and pastures [8,9,10]. Therefore, wild animals may act as a reservoir for PTB. Sharing pastures and water at the wildlife-livestock interface is considered a risk factor for infection transmission [11]. This also might extend the environmental persistence of the disease [12]. Moreover, MAP has been investigated for a possible link with Crohn’s disease and related inflammatory bowel diseases, as well as many other human conditions [13,14]. Although the role of MAP in human disease remains under investigation, it is considered a pathogen of public health concern because of its potential cross-species transmission [15]. Because wild animals may serve as a reservoir for MAP, the occurrence of PTB in wildlife highlights the need for surveillance at the wildlife- livestock- environment interface.

Diagnosis of PTB is a challenge because of the pathogen’s characteristics and prolonged course, which leads to different disease stages and, consequently, variations in immune response that also pose a challenge for diagnostic methods and techniques. Faecal culture is the gold standard method, but it requires a long incubation time to obtain growth [16]. Although immunodiagnostic assays such as ELISA have been successfully applied for PTB surveillance in some captive wild species with promising results [17], their widespread application should be preceded by thorough species-specific evaluation. To select diagnostic tests for free-ranging wildlife, several factors need to be considered, including animal welfare concerns associated with capture and handling, sample quality, and, most importantly, the availability of species-specific validation [18,19]. Most commercial ELISA kits have been developed for domestic livestock and might exhibit reduced diagnostic performance in wildlife due to variations in immunoglobulin structure, antibody responses, the lack of validated cut-off values, and the limited availability of reference sera [16,20]. Furthermore, ELISA has limited sensitivity during the prolonged subclinical stage of MAP infection and might be affected by cross-reactivity with environmental mycobacteria. Therefore, immunodiagnostic tests such as ELISA can provide valuable insights for PTB surveillance when appropriately validated for the target species [21]. However, they are recommended as complementary assays rather than standalone diagnostic tools for investigating PTB in general. Whenever applicable, positive or inconclusive findings should be confirmed using other diagnostic methods [17,21]. Molecular-based assays, such as polymerase chain reaction (PCR) and recombinase polymerase-aided amplification (RAA), offer a good alternative to culture; yet faecal samples remain the most difficult specimens for DNA extraction and amplification [22,23,24]. The RAA assay, combined with the rapid DNA extraction protocol, is a simple, rapid test with a sensitivity comparable to real-time PCR [23].

Globally, tourism and wildlife-related activities are important sources of livelihood for millions of people. In many African countries, particularly for some local communities, wildlife products represent a main source of income, provide career opportunities, and support conservation programmes at the national economy level. The emergence and transmission of zoonotic pathogens within wildlife populations may endanger this sector by affecting wildlife health and reducing tourist confidence. Consequently, understanding and managing zoonotic disease risk is important for both public health and sustainability of wildlife tourism in Africa [6,25].

Data on prevalence and epidemiology of PTB, along with its causative agent, were reported in Sudan in domestic animals including cattle (8.5% seroprevalence) and small ruminants (8.5% seroprevalence) [26,27,28,29,30,31,32], as well as in camels (unpublished data) and even in humans [13]. In spite of this, to date, there are no reports about the disease or its aetiology in wildlife.

The present study aimed to generate baseline data on paratuberculosis in wildlife in the Sudan. Here we provide evidence of the disease in wild animals by detecting MAP DNA in the faeces of some wild ruminants in one of the main biosphere reserves in the country.

## 2. Materials and Methods

### 2.1. Area of Study

**Figure 1:**
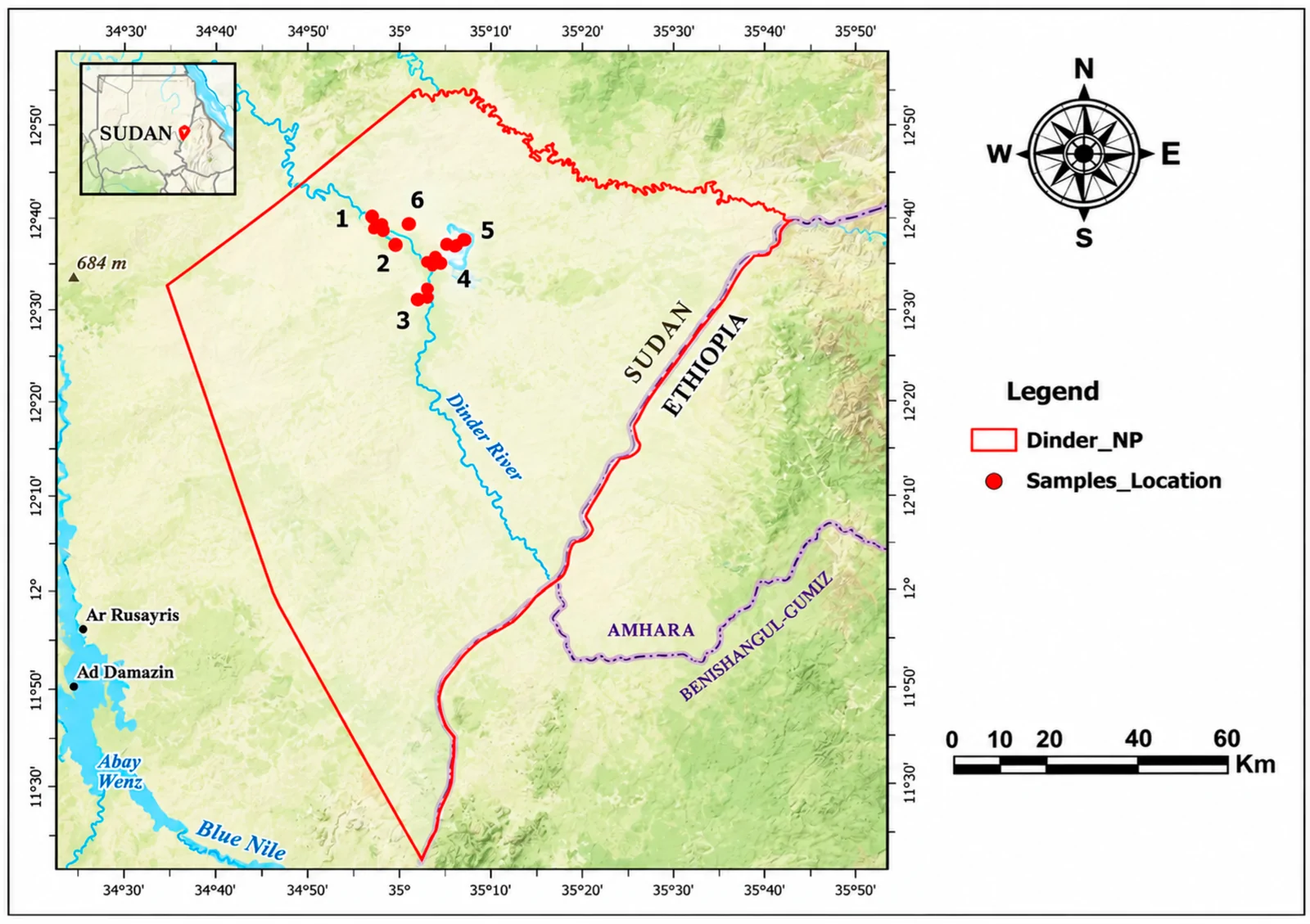
The Dinder National Park location and boundaries, with the sampling points marked with red spots and numbers: (1) Musa, (2) Gerarissa, (3) Berkat Tamasih, (4) Abdu Algani, (5) Rass Amir, and (6) Ein Alshemis. This map was created by ArcGIS Pro 3.6.

**Figure 2:**
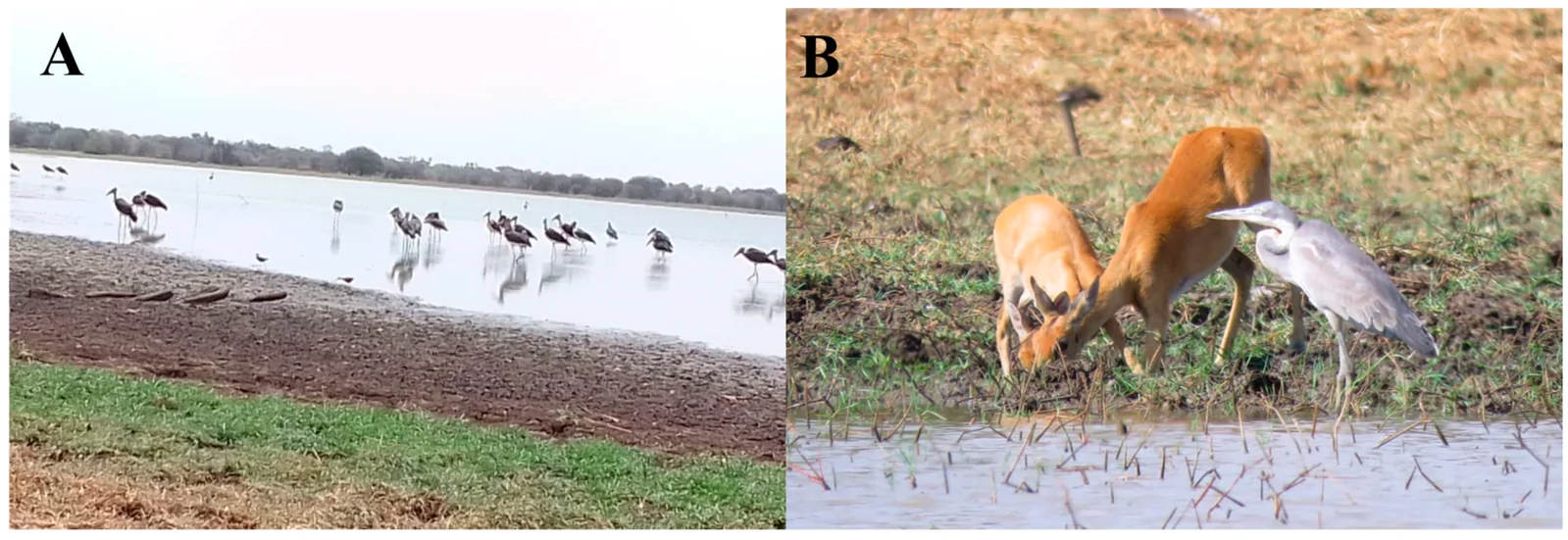
(**A**) The Rass Amir Pond (or Maya), one of the sampling areas; the wild birds appearing in the photo are marabou storks (*Leptoptilos crumeniferus*). The far side of the picture shows the trees surrounding the pond. (**B**) The wild ungulate, oribi (*Ourebia ourebia*), and a wild bird, the grey heron (*Ardea cinerea*), sharing the water source in the Dinder National Park. The photos are courtesy of Dr. Omer M. Meina.

The Dinder National Park (DNP) is located on either side of the River Dinder, bordered to the north by the River Rahad, between Latitudes 12°50′ and 11°45′ N and Longitudes 36°00′ E and 34°30′ (Figure 1), approximately 436 kilometers southeast of Khartoum State, Sudan, where it shares a 75 km long border with the Ethiopian Alitash National Park [33]. The Dinder National Park is one of Sudan’s largest protected ecosystems, hosting 27 species of large mammals, such as buffaloes, antelopes and warthogs, and about 250 bird species, including resident, local migrants, and pale arctic migrant birds; some of these mammals and birds are endangered species [34,35]. The park experiences two distinct climatic seasons: a mild dry season from December to April with a maximum temperature of 30 °C, and a hot, humid, and rainy season from May to November, with a maximum temperature of 44 °C [36]. Three main ecosystems mark the vegetation in the DNP: the riverine ecosystem, the Mayas ecosystem, and the *Vachellia seyal*–*Balanites aegyptiaca* ecosystem [37]. The Mayas (water ponds) wetlands (Figure 2A,B) are one of the DNP’s primary habitats and its most distinct feature [37]. The floodplain wetland is connected to over 40 Mayas found on both banks of the Dinder and the Rahad rivers. People from about 40 villages move in and out of the DNP as part of their animal grazing activity; about 10% of them live inside the park. Movement of humans together with their livestock across the protected areas during the dry season is common, thus increasing the possibility of disease transmission between wild and domestic animals [33,36].

### 2.2. Sampling Sites

For the collection of faecal samples, six sites, namely Abdu Algani, Ein Alshemis, Berkat Tamasih, Gerarissa, Rass Amir, and Musa (Figure 2A), were selected as the major water ponds in the DNP. These ponds are the main aggregation points where wildlife and occasionally domestic animals commonly congregate, particularly during the dry season when different animal species interact because available water sources are limited. The selected ponds were the most accessible water sources within the park; therefore, they are logistically suitable for the sampling. Moreover, they are located near the areas where PTB had previously been reported.

### 2.3. Sample Size

The sample size was calculated according to Cochran’s formula [38] as follows:

Sample size = Z^2^ × p × (1 − p)/C^2^

A 1.645 Z-value corresponding to a 90% confidence level was applied to obtain the minimum number of samples for an initial assessment of MAP detection, considering the practical constraints associated with wildlife sampling. Because no information about PTB prevalence in wild animals is available, an expected 50% prevalence (p) was considered, according to Cochran’s recommendation. This conservative estimate gives the largest possible sample size, thereby enhancing statistical power. A 5% margin of error (C) was chosen to ensure accurate prevalence estimation. Based on this calculation, the minimum required sample size was 271.

### 2.4. Sample Collection

The sampling time was chosen based on the observed wildlife activity during the sampling period and taking into consideration the feeding behaviour of the animals. The collection sites were visited in the morning, when animals gathered around the ponds. Animals were observed until they left the area, then fresh faecal samples were collected randomly. The sampling was conducted to maximize representation of different individuals within each species by considering differences in animal presence and faeces location. Although individual animals could not be identified, practical measures were taken to reduce the likelihood of repeated sampling from the same individual by collecting samples from different defecation spots and sampling each site only once during the visit. Despite these precautions, repeated sampling from the same individual animal cannot be completely excluded. Hand-grip faeces or faecal pellets amounting to about 100 mL of apparently fresh faeces were collected and placed in a labelled sterile plastic container. New medical examination gloves were used for each sample. Immediately after collection, the samples were maintained in an icebox at about 4 °C and transferred to the laboratory as soon as possible. Upon arrival, the samples were stored at −20 °C until processing.

### 2.5. Identification of the Animals’ Faeces

Relating the faeces to the animal species was made possible by watching the place while each animal species group gathered around the pond, in addition to the animal’s footprint and the faeces morphology, as guided by Stuart and Stuart [39].

### 2.6. The Animal Species

In total, 274 faecal samples were collected; they belonged to five wild ungulates: 71 reedbuck (*Redunca redunca*), 71 waterbuck (*Kobus defasa*), 54 oribi (*Ourebia ourebia*), 46 warthogs (*Phacochoerus aethiopicus*) and 32 African buffalo (*Syncerus caffer*).

### 2.7. Vegetation Features

The vegetation composition surrounding each sampling site (pond/Maya) was identified through direct observation; the trees and shrub species were recorded and then categorized into four patterns (A–D) based on the composition, as shown in Table 2. These patterns represented variations in dominant vegetation communities, with each pattern characterized by the presence and relative predominance of specific tree and shrub species. The vegetation patterns were subsequently evaluated as potential environmental factors associated with MAP DNA detection.

### 2.8. Processing of Samples

#### 2.8.1. DNA Extraction

Using the SwiftX kit DNA (Xpedite, Munich, Germany), DNA was extracted from the faecal samples following the manufacturer’s instructions [23] with some modifications. The samples were first processed to pellet the bacterial content as follows: approximately 3 g of faecal sample was suspended in 4 mL of distilled water, then the faecal suspension was mixed vigorously and allowed to stand for 5 min at room temperature to settle. The supernatant was then transferred to a new plain tube and centrifuged for 10 min. The supernatant was discarded again, and about 100 mg of the resulting faecal sediment was transferred to a Precelly’s SK 38 tube (Bertin Corp., Rockville, MD, USA); thereafter, 500 µL of lysis buffer and 60 µL of magnetic beads were added. The tubes were vortex-mixed for 3 s, then incubated in a heat block at 95 °C for 15 min, with mixing every 3 min. The tubes were then transferred to a magnetic rack and allowed to stand for 2–3 min. Subsequently, 10 µL from the clear solution against the magnet was collected and diluted with 40 µL of nuclease-free water. The resulting solution was used as the DNA template for the RAA assay targeting MAP IS900.

#### 2.8.2. Recombinase-Aided Amplification (RAA) Assay

A recombinase-aided amplification assay (RAA; QT Biotech Co., Ltd., Wuxi, China), essentially a recombinase polymerase amplification assay (RPA) [24]) targeting the IS900 of MAP was used to test the samples for the presence of MAP DNA. A mix containing 5 µL DNA, 29.5 µL rehydration buffer, 2.1 µL (10 pmol/µL) of the forward primer (5′-CGTGGACGCCGGTAAGGCCGACCATTACTGCATGG-3′) and reverse primer (5′-CGCCGCAATCAACTCCAGCAGCGCGGCCTC-3′), and 2 µL of exo probe (5′-ACGCCGGTAAGGCCGACCATTACTGCATGGTQTFTAACGACGACGCGCA-3′) was added to the freeze-dried reaction pellets. The reaction was performed at a constant temperature (42 °C) for 15 min and monitored using the TubeScanner (TwistDx TS1 device, Cambridge, UK). In each run, a molecular standard (DNA string with sequence corresponding to the target MAP IS900 region synthesized by GeneArt Life Technologies, Carlsbad, CA, USA) and nuclease-free water were used as positive and negative controls, respectively. The fluorescence cut-off on the TubeScanner screen is defined as the mean fluorescence of the negative control. A positive result is determined when the fluorescence exceeds this threshold within the validated time to threshold of 7 min [24]. Invalid runs, in which the controls did not perform as expected, were excluded from the analysis and repeated before sample results were considered valid; the final results were based on the repeated runs.

### 2.9. Statistical Analysis

True detection rate estimates of MAP DNA from RAA results were calculated using the EPITOOLS [40] at a 95% confidence level. True detection rate is the actual proportion of positive samples within the total population. To increase the accuracy of the disease prevalence MAP DNA detection estimate, the calculation included the assay’s 89.5% sensitivity and 100% specificity [24] to account for the false-positive and false-negative results.

## 3. Results and Discussion

**Figure 3:**
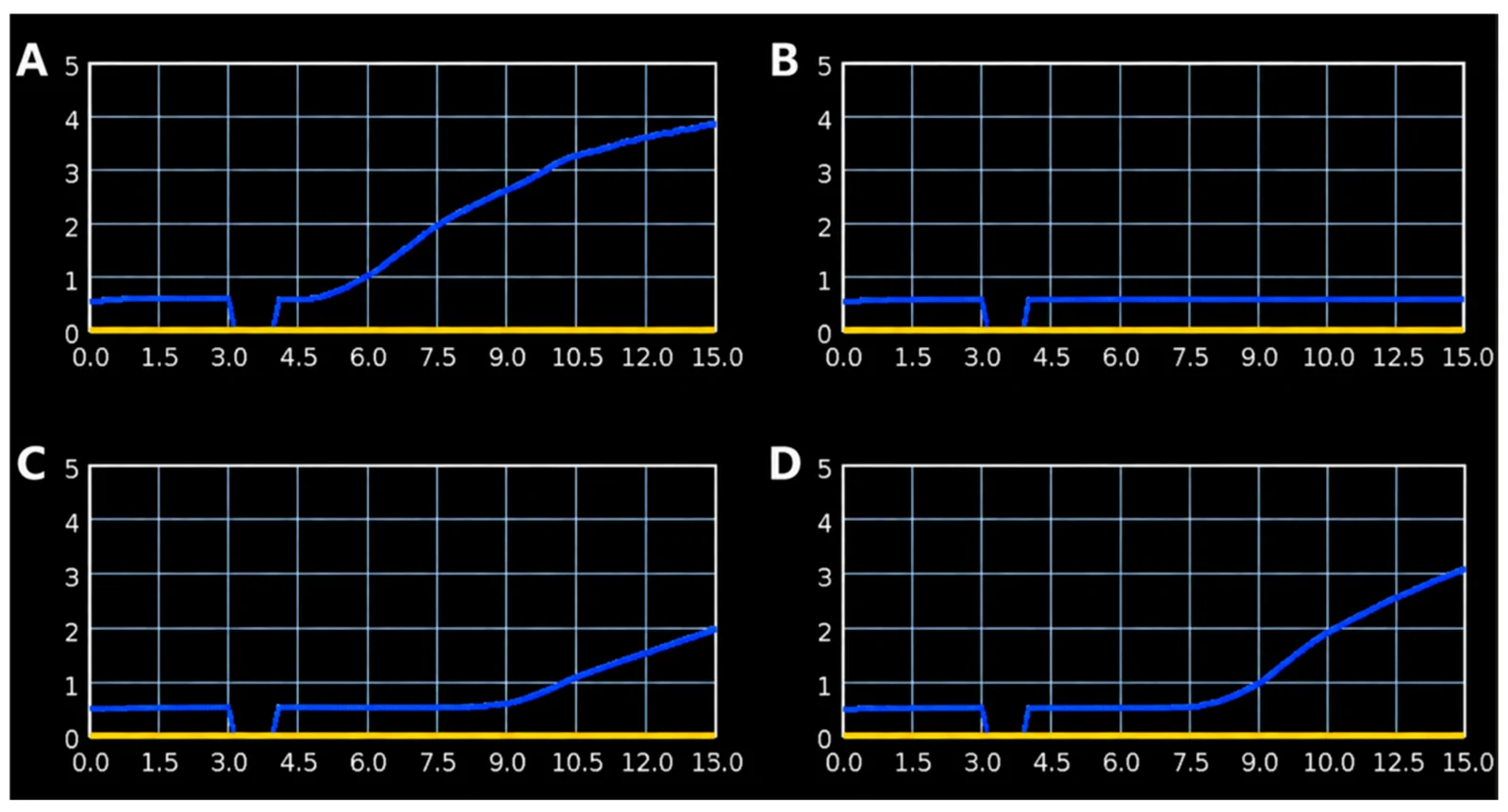
Representative fluorescence amplification curves for the recombinase-aided amplification (RAA) assay used for detecting *Mycobacterium avium* subspecies *paratuberculosis* DNA in faecal samples from wild animals. The fluorescence curves demonstrate real-time amplification of MAP DNA during the RAA reaction. Samples showing a characteristic increase in fluorescence that crossed the assay threshold within the prescribed reaction time 7 min, interpreted as positive, whereas samples showing no amplification signal were interpreted as negative. (**A**): positive control showing the expected amplification curve; (**B**): negative control showing no amplification; (**C**,**D**): represent positive samples. The X-axis represents the time, while the Y-axis represents the signal intensity. The absence of a signal between minutes 3.0 and 4.5 is caused by the mixing step, when the sample is removed from the device and vortexed.

Paratuberculosis (PTB) is an untreatable, debilitating infectious disease that affects both domestic and wild ruminant and non-ruminant species [9]. Although it has been reported in domestic ruminants in the Sudan [28,29,30,31,32,41,42], no reports on it in wildlife are available. This study investigated the presence of MAP DNA in faecal samples of wild animals in the Dinder National Park (DNP) to provide baseline data on PTB in wildlife in the Sudan. A total of 274 faecal samples were collected from five wild animal species belonging to *Bovidae* and *Suidae* families and tested for MAP DNA. The apparent detection rate of MAP DNA in the samples was 37.9% (104/274), while the estimated true detection rate calculated using the diagnostic test sensitivity (89.5%) and specificity (100%) was 42.4%. Previous studies from other parts of the world documented MAP presence in the faeces of wild animals in both free-range populations and animals maintained in zoological parks, suggesting a broad distribution of PTB across various wildlife settings [9,43,44]. The presence of MAP in the faeces of wild animals in this study has been shown by MAP DNA detection using a real-time recombinase-aided amplification (RAA) assay (Figure 3). Similar Molecular detection of MAP was reported from Italy [45] and Poland [12]. MAP was detected by real-time PCR in 5.34% (7/131) of faecal samples obtained from asymptomatic zoo ruminants and non-ruminants in Poland [12] and by quantitative PCR in the faeces of 60% (6/10) of an endangered Scimitar-horned *Oryx* species in Italy, most of which were showing signs of PTB and then necropsied [45]. Although the sensitivity and specificity of the current assay are comparable with those of real-time PCR [24], a direct comparison between the results of this study and those of the mentioned studies is not possible. In Polish and Italian studies, the animals were kept in captivity, while the animals in this study were free-living in their natural habitat. Also, the sample size in the Italian study and the stage of the disease were different.

Our findings show MAP DNA detection rates among the samples assigned to their animal species as follows: 39.4% (28/71), 39.1% (18/46), 38.0% (27/71), 37.5% (12/32) and 35.2% (19/54) in Reedbuck, Warthog, Waterbuck, African Buffalo and Oribi, respectively (Table 1). It is worth mentioning that Warthog, Oribi and Reedbuck are the most abundant wild animals inhabiting the DNP [46,47]. Therefore, these results suggest a wide presence of the organism among wild species and in the DNP. Several reports about MAP infection have been made in many wild animals worldwide, but PTB or MAP was not reported in some species in the current study. In Korea [48], MAP DNA was detected in 8.9% (16/180) of faecal samples collected from free-ranging wild boar (*Sus scrofa*). Also, MAP DNA was reported in 25 free-ranging North American bison (*Bison bison*) that exhibited clinical signs consistent with PTB in India [49]. A captive Mishmi takin (*Budorcas taxicolor taxicolor*) demonstrated high susceptibility to MAP when PTB appeared as an outbreak in a zoo in Scotland [50]. These findings highlight the potential role of various wild animal species in the epidemiology and environmental circulation of this pathogen worldwide. In fact, they show the significance of broader disease monitoring systems that integrate wildlife into PTB surveillance to better understand transmission dynamics of the disease. Such an approach would also develop rapid detection techniques and set more effective methods to reduce the spread of MAP in both wild animals and livestock.

The reported prevalence of PTB in free-ranging wild animals is generally lower and more sporadic than in livestock [51]; however, shared habitats and environmental contamination might facilitate MAP exposure and increase the potential disease transmission between wildlife and domestic herds [52]. Local communities inside the park and its surroundings, as well as pastoralists, utilize the DNP for various needs, including grazing their livestock [33], thus making co-grazing or grazing the same pasture by both domestic and wild animals unavoidable. As several species share the same pastures and water resources, opportunities for interspecies transmission of MAP are potentially high. Similar scenarios have been observed in Germany [53] and New Zealand [54], where co-grazing systems were shown to facilitate cross-species exposure to MAP. Therefore, free-grazing practices may contribute to disease prevalence.

Concerns about disease transmission from wildlife to other animals are frequently raised. However, proving such transmission under field conditions remains challenging. The MAP’s long incubation period may facilitate subtle spread of the infection from subclinical MAP shedders. Other potential sources of infection include vertical and airborne transmission, as well as environmental contamination [55]. As MAP does not replicate outside the host [56], and the infection is non-species specific, the range of disease transmission could expand to include other animal species [57]. Documenting the MAP DNA presence in the faeces of wild animals inhabiting the DNP highlights the need for further studies on PTB epidemiology and population genetics in these species. Molecular genotyping assays could assess the genetic relatedness among MAP strains [58] that are potentially circulating in the DNP and identify the potential transmission routes between wildlife and other susceptible animals. Interaction between livestock, humans, and wild animals is known in the DNP [59], and MAP infection has been recently documented in livestock in some states sharing borders with the DNP. MAP seroprevalence (by ELISA) and MAP molecular prevalence (by RAA) were reported as 54.2% and 24.9%, respectively, in cattle [31], and as 8.50% and 41.8%, respectively, in small ruminants [32]; therefore, cross-interface transmission cannot be excluded and warrants investigation. Investigations on MAP genotypes circulating in wildlife populations in the DNP and domestic animals around the DNP may provide insights into disease transmission at this interface.

Wild resident or migrant birds are an important population entity among DNP inhabitants, and frequently share grazing areas with wild animals (Figure 2B). Although wild birds are considered mechanical carriers rather than true MAP hosts [60,61], future studies should investigate the presence of MAP in wild birds’ faeces in the DNP to know whether they have a role in PTB transmission and environmental contamination. Furthermore, the birds’ movement and behaviour might facilitate MAP dissemination across spatially distinct areas within and around the park.

**Figure 4:**
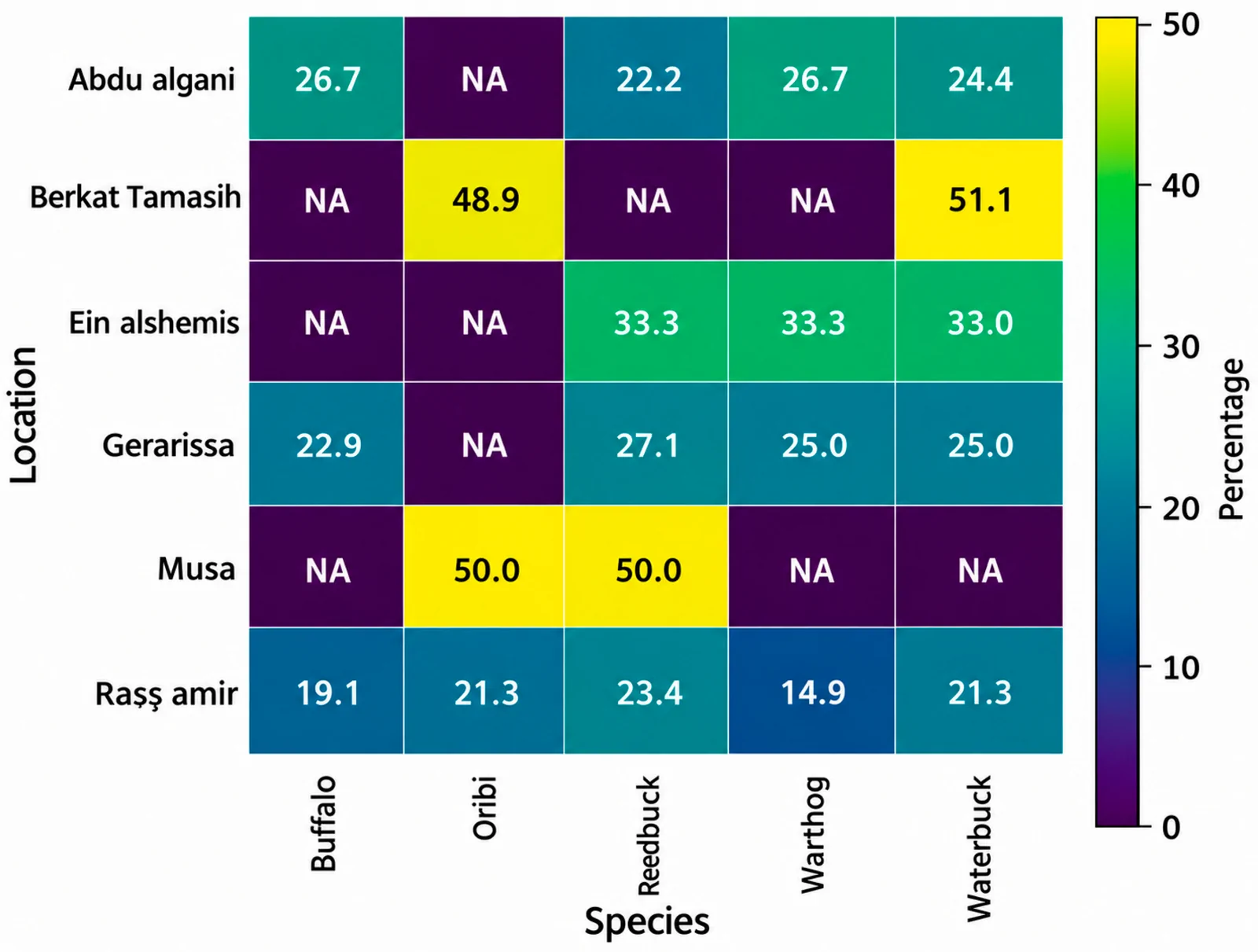
Spatial representation of MAP DNA detection rate in faecal samples of wild animals in gathering areas (ponds/Mayas) in the Dinder National Park using a recombinase- aided amplification (RAA) assay. NA indicates that no faecal samples of these species were found around the pond.

In the DNP, wild animals gather in specific locations during the dry season, where they share water and pasture; these locations may create hotspots for MAP exposure and disease transmission. Our findings suggest that the MAP DNA detection rate within the DNP varies from one area to another (Figure 4). The lowest MAP DNA detection was recorded in the ponds Gerarissa and Ras Amir, while Abdu Algani, Ein Alshemis and Musa respectively recorded the highest detection rates (Table 1). The high proportion of potentially infected wild animals in these areas might facilitate MAP transmission, thereby contributing to the persistence of PTB in the DNP.

Pasture density and water availability are key factors determining the number of animals that can gather and, consequently, the number exposed to the disease.

The ecological niche might contribute to variation in MAP DNA detection rates among sampling sites. During sample collection, we identified various trees and shrubs characteristic of African Savannah and dry woodland ecosystems in the vegetation around the ponds. Vegetation characteristics were included as an ecological variable, as vegetation structure around water sources can influence animal activity, grazing behaviour, and habitat utilization, which may subsequently affect the deposition and persistence of MAP in the environment [62]. Although other environmental factors, including soil properties, water quality parameters, biodiversity indices, and animal density, may also contribute to MAP occurrence and persistence [56], vegetation was selected as an observable field-based habitat characteristic during the sampling. The trees and shrubs could be grouped into four vegetation patterns (Table 2). MAP DNA detection rates varied among vegetation patterns. The highest MAP DNA detection rate (55.6%) was observed in pattern B, which is characterized by *Vachellia seiberiana* and *Acacia polyacantha*, followed by pattern C (40.9%). While pattern D showed a relatively low detection rate (31.1%), pattern A, characterized by *Ziziphus abyssinica* and *Crateva adansonii*, showed the lowest rate (23.2%) (Table 3); both patterns (A and D) are characterized by the shrub *Grewia villosa*. Although these vegetation pattern differences were not quantified systematically in this study, they are noteworthy and require further investigations to assess whether an association between the vegetation around the ponds and the MAP DNA detection rate can be established statistically. Animal species composition around the ponds might also affect MAP DNA detection rate because of the grazing preferences of the animals [63,64]. Many animals feed on the fruits of *Ziziphus abyssinica* and *Crateva adansonii* at specific times, both of which have antimicrobial properties [65,66]. Whether these two plants, as well as *Grewia villosa*, have an antimycobacterial effect in animals that feed on them or modulate their gut microbiome, resulting in lower MAP burden and potentially reduced MAP shedding into the environment, is unclear and requires further investigation. The animal composition and grazing preferences, in addition to other factors such as the size of the grazing area and soil type, were not investigated in this study. The complexity of these factors, along with the vegetation around the ponds, might affect the MAP DNA detection rate.

Because of the PTB nature and MAP characteristics, more than one diagnostic test is needed to identify and confirm a MAP-infected animal [4], as available diagnostics vary in sensitivity and specificity [62]. Although ELISA is a known cost-effective PTB diagnostic test [67], it was not applicable in this study because capturing and handling free-ranging wild animals is a great challenge. In addition, many authors raised concerns about its application in wild animals [16,18,19,20].

Molecular techniques have become more convenient, offering detection and high sensitivity in a relatively short time [68]. The RAA assay used in this study showed a high MAP DNA detection rate among the tested samples. It had previously been used in a PTB survey in zoo animals in Germany [67], but none of the faecal samples showed MAP DNA positive results, which was confirmed by real-time PCR. Moreover, the same RAA assay was used alongside ELISA to investigate MAP infection in ruminants in Sudan and Uganda [29,30,31,32,69]. The results showed that the RAA assay has a fair sensitivity for MAP detection and provides convenient estimates of disease prevalence comparable to ELISA results [30,69]. Since RAA has a high sensitivity and specificity [24], it would be a useful tool for providing general insights into disease prevalence in animals. Indeed, when integrated into the mobile suitcase lab [70], it is practical in wildlife surveys. Moreover, when coupled with a DNA rapid extraction protocol and using faecal samples to reduce the challenges in sampling free-range wildlife as done in this study, it shortens the time to obtain results compared with other molecular techniques.

### Limitations of the Study

Several limitations affected the study design and sampling and have influenced how well the diversity of wild animal species in the DNP was represented. Sampling free-ranging wild animals is complicated because of the DNP’s large size and the limited accessibility to many areas most of the year. Consequently, sample collection was restricted to the major water ponds (Mayas), where animals gathered during the dry season. Moreover, the behaviour of some wild species limited sampling to animals that gathered around the ponds in the early morning, where only fresh faecal samples were collected. As sampling from the same animal could not be completely excluded despite the great care to avoid it, our species-specific detection rate estimates might be affected. MAP DNA-positive samples were not confirmed by another assay or by culture to assess viability of the bacteria.

## 4. Conclusions

To the best of our knowledge, this is the first molecular evidence of MAP in wildlife-associated faecal samples in Sudan and probably one of the first surveys of MAP in wild animals in Africa. The high detection rate of MAP DNA among wild animals is a concern for the productivity of in-contact farmed animals and biodiversity and conservation of endangered species in the DNP.

Future studies and surveillance efforts should be designed to investigate and confirm PTB incidence in susceptible and endangered wild animals. Such studies would enhance our understanding of MAP transmission dynamics and epidemiology and help design disease control programmes. Furthermore, applying molecular typing and molecular epidemiology approaches will provide valuable insights into the genetic diversity across wild species.

## Figures and Tables

**Table 1:** Frequency of detection of *Mycobacterium avium* subspecies *paratuberculosis* DNA among faecal samples of wild animal species across the six major animal gathering areas and their relative location in the Dinder National Park.

Water Pond (Maya)	Samples (n)	MAP-Positive n (%)	Reedbuck (*Redunca* *redunca*)	Waterbuck (*Kobus* *defasa*)	African Buffalo (*Syncerus caffer*)	Oribi (*Ourebia ourebi*)	Warthog (*Phacochoerus aethiopicus*)
Ein Alshemis	45	29 (64.4)	10/15	10/15	-	-	9/15
Abdu Algani	45	21 (46.7)	5/10	4/11	6/12	-	6/1
Musa	44	18 (40.9)	9/22	-	-	9/22	-
Berkat Tamasih	45	14 (31.1)	-	6/23	-	8/22	-
Gerarissa	48	11 (22.9)	2/13	3/12	3/11	-	3/1
Rass Amir	47	11 (23.4)	2/11	4/10	3/9	2/10	0/7
	274	104 (37.97)	28/71	27/71	12/32	19/54	18/46

**Table 2:** Common vegetation features around the six major water ponds (Mayas) in the Dinder National Park.

Patterns ID	Pond	Tree		Shrub	
		Local Name	Scientific Name	Local Name	Scientific Name
Pattern A	Rass Amir Gerarissa	Higleig	*Balanites aegyptiaca*	Halio	*Grewia villosa*
		Habil	*Combretum aculeatum*	Najeel/Nagila	*Cynodon dactylon*
		Talh	*Vachellia seyal* var. *seyal*	Adar	*Surgum sudanensis*
		Sidir	*Ziziphus abyssinica*	Arkala	*Ipomoea aquatica*
		Dabkar	*Crateva adansonii*	Khodra	*Corchorus olitorius*
		Abo khameira	*Piliostigma reticulatum*		
Pattern B	Ein Alshemis Abdu Algani	Higleig	*Balanites aegyptiaca*	Najeel/Nagila	*Cynodon dactylon*
		Habil	*Combretum aculeatum*	Adar	*Surgum sudanensis*
		Talh	*Vachellia seyal* var. *seyal*	Arkala	*Ipomoea aquatica*
		Kuk	*Vachellia seiberana*	Khodra	*Corchorus olitorius*
		Kakamout	*Acacia polyacantha*		
Pattern C	Musa	Higleig	*Balanites aegyptiaca*	Adar	*Surgum sudanensis*
		Habil	*Combretum aculeatum*	Arkala	*Ipomoea aquatica*
		Talh	*Vachellia seyal* var. *seyal*	Najeel/Nagila	*Cynodon dactylon*
		Abu khameira	*Piliostigma reticulatum*	Difera	*Echinochloa colonum*
				Ramtouk	*Xanthium brasilicum*
				Tarfa	*Tamarix aphylla*
Pattern D	Berkat Tamasih	Talh	*Vachellia seyal* var. *seyal*	Adar	*Surgum sudanensis*
		Kuk	*Vachellia seiberana*	Halio	*Grewia villosa*
		Dom	*Hyphaene thebaica*	Ramtouk	*Xanthium brasilicum*

**Table 3:** Detection frequency of *Mycobacterium avium* subspecies *paratuberculosis* DNA at different sampling sites in the Dinder National Park as grouped according to the vegetation pattern.

Vegetation Pattern	MAP-DNA Positive	MAP-DNA Negative	Total
Pattern A	22 (23.2%)	73	95
Pattern B	50 (55.6%)	40	90
Pattern C	18 (40.9%)	26	44
Pattern D	14 (31.1%)	31	45
Total	104	170	274

## Data Availability

All data are presented in the manuscript, and requests for additional information can be made to the corresponding authors.

## References

[1] Meles D.K., Mustofa I., Khairullah A.R., Wurlina W., Mustofa R.I., Suwasanti N., Akintunde A.O., Putra S.W., Kusala M.K.J., Moses I.B. (2024). A comprehensive review of paratuberculosis in animals and its implications for public health. Open Vet. J..

[2] Whittington R., Donat K., Weber M.F., Kelton D., Nielsen S.S., Eisenberg S., Arrigoni N., Juste R., Saez J.L., Dhand N. (2019). Control of paratuberculosis: Who, why and how. A review of 48 countries. BMC Vet. Res..

[3] Fecteau M.E., Behr M.A., Stevenson K., Kapure V. (2020). Paratuberculosis in Cattle. Prartuberculosis. Organism, Disease, Control.

[4] Imada J., Kelton F.D., Barkema H.W., Behr M.A., Stevenson K., Kapur V. (2020). Epidemiology, Global Prevalence and Economics of the infection. Partuberculosis: Organism, Disease, Control.

[5] Manning E.J. (2001). Mycobacterium avium subspecies paratuberculosis: A review of current knowledge. J. Zoo. Wildl. Med..

[6] Kennedy D.J., Benedictus G. (2001). Control of Mycobacterium avium subsp. paratuberculosis infection in agricultural species. Rev. Sci. Tech..

[7] Daniels M.J., Hutchings M.R., Beard P.M., Henderson D., Greig A., Stevenson K., Sharp J.M. (2003). Do non-ruminant wildlife pose a risk of paratuberculosis to domestic livestock and vice versa in Scotland?. J. Wildl. Dis..

[8] Florou M., Leontides L., Kostoulas P., Billinis C., Sofia M., Kyriazakis I., Lykotrafitis F. (2008). Isolation of Mycobacterium avium subspecies paratuberculosis from non-ruminant wildlife living in the sheds and on the pastures of Greek sheep and goats. Epidemiol. Infect..

[9] Stevenson K., Alvarez J., Bakker D., Biet F., de Juan L., Denham S., Dimareli Z., Dohmann K., Gerlach G.F., Heron I. (2009). Occurrence of Mycobacterium avium subspecies paratuberculosis across host species and European countries with evidence for transmission between wildlife and domestic ruminants. BMC Microbiol..

[10] Čurlík J., Lazár P., Iglódyová A., Barbušinová E., Šmiga Ľ., Novotný J., Mojžišová J., Ondrejková A., Hromada R., Konjević D. (2020). Detection of Mycobacterium avium subsp. paratuberculosis in Slovakian wildlife. Pol. J. Vet. Sci..

[11] Rangel S.J., Paré J., Doré E., Arango J.C., Côté G., Buczinski S., Labrecque O., Fairbrother J.H., Roy J.P., Wellemans V. (2015). A systematic review of risk factors associated with the introduction of Mycobacterium avium spp. paratuberculosis (MAP) into dairy herds. Can. Vet. J..

[12] Bruczyńska M., Didkowska A., Brzezińska S., Nowak M., Filip-Hutsch K., Kalicki M., Augustynowicz-Kopeć E., Anusz K. (2023). Mycobacterium aviums subspecies paratuberculosis in asymptomatic zoo herbivores in Poland. Animals.

[13] Elmagzoub W.A., Idris S.M., Isameldin M., Arabi N., Abdo A., Ibrahim M., Khan M.A.A., Tanneberger F., Bakhiet S.M., Okuni J.B. (2022). Mycobacterium avium subsp. paratuberculosis and microbiome profile of patients in a referral gastrointestinal diseases centre in the Sudan. PLoS ONE.

[14] Mitchell R.M., Beaver A., Knupfer E., Pradhan A.K., Fyock T., Whitlock R.H., Schukken Y.H. (2019). Elucidating Transmission Patterns of Endemic Mycobacterium avium subsp. paratuberculosis Using Molecular Epidemiology. Vet. Sci..

[15] Sechi L.A., Dow C.T. (2015). Mycobacterium avium ss. paratuberculosis Zoonosis—The Hundred Year War—Beyond Crohn’s Disease. Front. Immunol..

[16] Roller M., Hansen S., Knauf-Witzens T., Oelemann W.M.R., Czerny C.P., Abd El Wahed A., Goethe R. (2020). Mycobacterium avium Subspecies paratuberculosis Infection in Zoo Animals: A Review of Susceptibility and Disease Process. Front. Vet. Sci..

[17] Pruvot M., Forde T.L., Steele J., Kutz S.J., Buck J.D., Meer F.v.d., Orsel K. (2013). The modification and evaluation of an ELISA test for the surveillance of Mycobacterium avium subsp. paratuberculosis infection in wild ruminants. BMC Vet. Res..

[18] Prieto J.M., Balseiro A., Casais R., Abendano N., Fitzgerald L.E., Garrido J.M., Juste R.A., Alonso-Hearn M. (2014). Sensitive and specific enzyme-linked immunosorbent assay for detecting serum antibodies against Mycobacterium avium subsp. paratuberculosis in fallow deer. Clin. Vaccine Immunol..

[19] Thomas J., Balseiro A., Gortazar C., Risalde M.A. (2021). Diagnosis of tuberculosis in wildlife: A systematic review. Vet. Res..

[20] Ochai S.O., Crafford J.E., Kamath P.L., Turner W.C., van Heerden H. (2023). Development of conjugated secondary antibodies for wildlife disease surveillance. Front. Immunol..

[21] Nielsen S.S., Toft N. (2008). Ante mortem diagnosis of paratuberculosis: A review of accuracies of ELISA, interferon-gamma assay and faecal culture techniques. Vet. Microbiol..

[22] Vansnick E., Vercammen F., Bauwens L., D’Haese E., Nelis H., Geysen D. (2005). A survey for Mycobacterium avium subspecies paratuberculosis in the Royal Zoological Society of Antwerp. Vet. J..

[23] Hansen S., Roller M., Alslim L.M.A., Bohlken-Fascher S., Fechner K., Czerny C.P., Abd El Wahed A. (2019). Development of Rapid Extraction Method of Mycobacterium avium subspecies paratuberculosis DNA from Bovine Stool Samples. Diagnostics.

[24] Hansen S., Schafer J., Fechner K., Czerny C.P., Abd El Wahed A. (2016). Development of a Recombinase Polymerase Amplification Assay for Rapid Detection of the Mycobacterium avium subsp. paratuberculosis. PLoS ONE.

[25] Chardonnet P., des Clers B., Fischer J., Gerhold R., Jori F., Lamarque F. (2002). The value of wildlife. Rev. Sci. Tech..

[26] Fawi M.T., Obeid H.M. (1964). A note on Johne’s disease among cattle in the Sudan. Bull. Epiz. Dis. Afr..

[27] Chaudhary A., Fawi M., Obeid H. (1964). Johne’s disease in goats in the Sudan. Vet. Rec..

[28] Elmagzoub W.A., Adam N.M., Idris S.M., Mukhtar M.E., Abdelaziz S.A., Okuni J.B., Ojok L., Abd El Wahed A., Eltayeb E., Gameel A.A. (2020). Seroprevalence of Mycobacterium avium subsp. paratuberculosis in Dairy Cattle in Khartoum State, Sudan. Vet. Sci..

[29] Elmagzoub W.A., Idris S.M., Elnaiem M.H.E., Mukhtar M.E., Eltayeb E., Bakhiet S.M., Okuni J.B., Ojok L., El Sanousi S.M., El Wahed A.A. (2024). Faecal microbial diversity in a cattle herd infected by Mycobacterium avium subsp. paratuberculosis: A possible effect of production status. World J. Microbiol. Biotechnol..

[30] Idris S.M., Ali E.A., Elmagzoub W.A., Okuni J.B., Mukhtar M.E., Ojok L., Eltayeb E., Abd El Wahed A., Eltom K.H., Gameel A.A. (2022). First Report on Ovine Paratuberculosis in the Sudan: Diagnosis Using Different Techniques. Animals.

[31] Idris S.M., Elmagzoub W.A., Mukhtar M.E., Okuni J.B., Ojok L., Abdalla E.M., Sanousi S.M.E., Amanzada A., Truyen U., Wahed A.A.E. (2025). Prevalence and molecular insights of Mycobacterium avium subsp. paratuberculosis in Sudanese Cattle: Implications for control and public health. Braz. J. Microbiol..

[32] Idris S.M., Elmagzoub W.A., Okuni J.B., Ojok L., Mukhtar M.E., Abdalla E.M., El Sanousi S.M., Amanzada A., Truyen U., El Wahed A.A. (2025). Paratuberculosis in small ruminants in the Sudan: Prevalence and risk factors. BMC Vet. Res..

[33] Geleto M.A., Kapp G., Sanjak E. (2023). Coping with local peoples’ livelihood dependence on the transboundary Dinder-Alatish National Parks in Sudan and Ethiopia from the Sudanese Perspective. Environ. Dev. Sustain..

[34] World Heritage Convention Dinder National Park. https://whc.unesco.org/en/tentativelists/6515/#:~:text=It%20is%20a%20prominant%20habitat,an%20international%20importance%20since%202005.

[35] Yousif R.A. (2012). Abundance, Mayas Preference and Distribution of Birds in Dinder National Park, Sudan. World’s Vet. J..

[36] Bauer H., Awad A., Sitotaw E., Sillero-Zubiri C. (2017). Follow-Up Visits to Alatash—Dinder Lion Conservation Unit: Ethiopia & Sudan.

[37] Elmardi M.E.M., Hassan M.M.A., Ibrahim M.E.A., Hamid A.A., Siddig A.A.H. (2025). Impact of Fire Frequency on Tree Diversity in Dinder National Park (DNP), Sudan Using Geospatial Technology. J. Geosci. Environ. Prot..

[38] Cochran W.G. (1977). Sampling Techniques.

[39] Stuart C., Stuart T. (2009). Mammals of East Africa.

[40] Sergeant E.S.G. Epitools Epidemiological Calculators. http://epitools.ausvet.com.au.

[41] Abbas B., Idris S.E.O., Burhan A. (1986). Isolation of M. paratuberculosis from goats in Sudan. Sudan. J. Vet. Sci. Anim. Husb..

[42] Abubaker A.M., El Sanousi S.M. (1975). A survey of Johne’s disease in Khartoum. Vet. Rec..

[43] Munster P., Fechner K., Volkel I., von Buchholz A., Czerny C.P. (2013). Distribution of Mycobacterium avium ssp. paratuberculosis in a German zoological garden determined by IS900 semi-nested and quantitative real-time PCR. Vet. Microbiol..

[44] Salgado M., Corti P., Verdugo C., Tomckowiack C., Moreira R., Durán K., Avilez C., Tejeda C. (2017). Evidence of Mycobacterium avium subsp. paratuberculosis (MAP) infection in huemul deer (*Hippocamelus bisulcus*) in patagonian fjords. Austral J. Vet. Sci..

[45] Pigoli C., Garbarino C., Ricchi M., Bonacina E., Gibelli L., Grieco V., Scaltriti E., Roccabianca P., Sironi G., Russo S. (2020). Paratuberculosis in Captive Scimitar-Horned Oryxes (Oryx dammah). Animals.

[46] (2004). Management Plan for Dinder National Park, Sudan.

[47] Mohammed A.A., Bauer H., Coals P., Meina O., Elsadig I., Ahmed R., Yahya E., Brema N., Ibrahim G.E., Mami A.M. (2023). Abundance of larger mammals in Dinder National Park, Sudan. Eur. J. Wildl. Res..

[48] Kim J.M., Ku B.K., Lee H.N., Hwang I.Y., Jang Y.B., Kim J., Hyun B.H., Jung S.C. (2013). Mycobacterium avium paratuberculosis in wild boars in Korea. J. Wildl. Dis..

[49] Yadav D., Singh S.V., Singh A.V., Sevilla I., Juste R.A., Singh P.K., Sohal J.S. (2008). Pathogenic ‘Bison-type’ Mycobacterium avium subspecies paratuberculosis genotype characterized from riverine buffalo (Bubalus bubalis) in North India. Comp. Immunol. Microbiol. Infect. Dis..

[50] Richardson H., Rhodes G., Henrys P., Sedda L., Weightman A.J., Pickup R.W. (2019). Presence of Mycobacterium avium Subspecies paratuberculosis Monitored Over Varying Temporal and Spatial Scales in River Catchments: Persistent Routes for Human Exposure. Microorganisms.

[51] Duarte L., Santos-Reis M., Cunha M.V. (2024). Widespread circulation and transmission risk of Mycobacterium avium subsp. paratuberculosis at the livestock-wildlife-environment interface in a Mediterranean agro-forestry farmstead. Environ. Pollut..

[52] Garbarino C., Nava M., Filippi A., Forti A., Russo S., Barsi F., Bianchi A., Filipe J., Arrigoni N., Pedrotti L. (2025). Contamination of alpine pastures by Mycobacterium avium subsp. paratuberculosis: Evaluation of cattle and red deer contribution through environmental fecal samples. Vet. Microbiol..

[53] Fritsch I., Luyven G., Kohler H., Lutz W., Mobius P. (2012). Suspicion of Mycobacterium avium subsp. paratuberculosis transmission between cattle and wild-living red deer (Cervus elaphus) by multitarget genotyping. Appl. Environ. Microbiol..

[54] Verdugo C., Pleydell E., Price-Carter M., Prattley D., Collins D., de Lisle G., Vogue H., Wilson P., Heuer C. (2014). Molecular epidemiology of Mycobacterium avium subsp. paratuberculosis isolated from sheep, cattle and deer on New Zealand pastoral farms. Prev. Vet. Med..

[55] Pal M., Rahman M.T. (2015). Mycobacterium avium subspecies paratuberculosis: An Emerging Bacterial Disease of Global Public Health Significance. Microbes Health.

[56] Whittington R.J., Marshall D.J., Nicholls P.J., Marsh I.B., Reddacliff L.A. (2004). Survival and dormancy of Mycobacterium avium subsp. paratuberculosis in the environment. Appl. Environ. Microbiol..

[57] Payan-Carreira R. (2013). Insights from Veterinary Medicine.

[58] Fawzy A., Zschock M., Ewers C., Eisenberg T. (2018). Genotyping methods and molecular epidemiology of Mycobacterium avium subsp. paratuberculosis (MAP). Int. J. Vet. Sci. Med..

[59] Hoven W.V., Nimir M.B. (2004). Recovering from conflict: The case of Dinder and other national parks in Sudan. War. Prot. Areas.

[60] Corn J.L., Manning E.J., Sreevatsan S., Fischer J.R. (2005). Isolation of Mycobacterium avium subsp. paratuberculosis from free-ranging birds and mammals on livestock premises. Appl. Environ. Microbiol..

[61] Seva J., Sanes J.M., Mas A., Ramis G., Sanchez J., Parraga-Ros E. (2022). Prevalence of Mycobacterium avium Subsp. paratuberculosis in Feral Pigeons (Columba livia) Associated with Difficulties Controlling Paratuberculosis in a Bovine Herd (Fighting Bull Breed). Animals.

[62] Kanga E.M., Ogutu J.O., Piepho H.-P., Olff H. (2023). Hippopotamus and livestock grazing near water points: Consequences for vegetation cover, plant species richness and composition in African savannas. Front. Ecol. Evol..

[63] Deng G.T., Demisse B.T. (2021). Feeding Preference and Habitat Association of Defassa Waterbuck (Kobus ellipsiprymnus defassa) in Nechisar National Park, Southern Ethiopia. Sci. World J..

[64] Pansu J., Guyton J.A., Potter A.B., Atkins J.L., Daskin J.H., Wursten B., Kartzinel T.R., Pringle R.M. (2019). Trophic ecology of large herbivores in a reassembling African ecosystem. J. Ecol..

[65] Mignanwandé Z.F., Johnson R.C., Hounkpatin A.S.Y., Boni G., Houndeton A.G., Houéto E.E., Kpètèhoto W.H., Amoussa M.O. (2020). Antibacterial Activities of the Ethanolic Extract of *Crateva adansonii* DC. (Capparidaceae) Harvested in Dassa-Zoumè in Central Bénin. Open J. Med. Microbiol..

[66] Mohammed N., Oloninefa S.D., Aisoni J.E., Fadayomi V.K., Sanusi S., Aliyu A. (2024). Phytochemical analysis and antibacterial activity of Crateva adansonii DC leaves and stem bark extracts against some pathogenic bacteria. Sci. World J..

[67] Roller M., Hansen S., Böhlken-Fascher S., Knauf-Witzens T., Czerny C.P., Goethe R., Abd El Wahed A. (2020). Molecular and Serological Footprints of Mycobacterium avium Subspecies Infections in Zoo Animals. Vet. Sci..

[68] Nielsen S.S., Behr M.A., Stevenson K., Kapur V. (2020). Immune-Based diagnosis of Paratuberculolsis. Paratuberculosis, Organism, Disease, Control.

[69] Ssekitoleko J., Ojok L., Omala S.K., Mukhtar M.E., Eltom K.H., Eltayeb E.S., Kankya C., Kisekka M., Truyen U., Czerny C.P. (2022). Molecular and serological survey of paratuberculosis in cattle in selected districts of Western Uganda. BMC Vet. Res..

[70] Abd El Wahed A., Weidmann M., Hufert F.T. (2015). Diagnostics-in-a-Suitcase: Development of a portable and rapid assay for the detection of the emerging avian influenza A (H7N9) virus. J. Clin. Virol..

